# Bevacizumab treatment for radiation brain necrosis: mechanism, efficacy and issues

**DOI:** 10.1186/s12943-019-0950-1

**Published:** 2019-02-07

**Authors:** Hongqing Zhuang, Siyu Shi, Zhiyong Yuan, Joe Y. Chang

**Affiliations:** 1 Department of Radiation Oncology, Peking University Third Hospital, Beijing, China; 2 Stanford University School of Medicine, Stanford, CA94305 USA; 3 Department of Radiotherapy, Tianjin Medical University Cancer Institute and Hospital, National Clinical Research Center for Cancer, Tianjin Key Laboratory of Cancer Prevention and Therapy, Tianjin, China; 4 Department of Radiation Oncology, Division of Radiation Oncology, the University of Texas MD Anderson Cancer Center, Houston, TX TX77054 USA; 5 Beijing, People’s Republic of China

**Keywords:** Bevacizumab, Radiation brain necrosis, Indication, Drug resistance

## Abstract

Vascular damage is followed by vascular endothelial growth factor (VEGF) expression at high levels, which is an important mechanism forradiation brain necrosis development. Bevacizumab alleviates brain edema symptoms caused by radiation brain necrosis through inhibiting VEGF and acting on vascular tissue around the brain necrosis area. Many studies have confirmed that bevacizumab effectively relieves symptoms caused by brain necrosis, improves patients’ Karnofsky performance status (KPS) scores and brain necrosis imaging. However, necrosis is irreversible, and hypoxia and ischemia localized in the brain necrosis area may easily lead to radiation brain necrosis recurrence after bevacizumab is discontinued. Further studies are necessary to investigate brain necrosis diagnoses, bevacizumab indications, and the optimal mode of administration, bevacizumab resistance and necrosis with a residual or recurrent tumor.

## Background

In 2007, Gonzalez J [1] first reported using bevacizumab treatment for radiation brain necrosis. Since then, many studies have confirmed that bevacizumab is an effective treatment for radiation brain necrosis [2–9].However, the sample size in most studies has been small, and many studies are case reports [10–12]; as a result, many questions remain unanswered. Herein, to provide a reference for researchers, we review the literature on using bevacizumab to treat radiation brain necrosis and summarize the mechanisms for, clinical efficacy of and current issues facing bevacizumab treatment of radiation brain necrosis.

### Mechanisms for bevacizumab treatment of radiation brain necrosis

Bevacizumab is used to treat radiation brain necrosis based on the mechanisms underlying radiation brain necrosis. Among many theories on radiation brain necrosis development, a vascular mechanism is widely accepted. Due to its effect on vascular tissue around a tumor, radiation causes vascular tissue damage followed by an oxygen diffusion disorder between the tissue and vessels and, subsequently, tissue hypoxia, which trigger increased expression of hypoxia-inducible factor (HIF)-1α. Next, tumor tissue hypoxia and elevated HIF-1α expression stimulates reactive astrocytes to secrete the pro-angiogenic factor VEGF. High levels of VEGF expression yield abnormal neovascularization, and the vessels formed lack a normal vessel structure and exhibit a disordered and fragile structure as well as high permeability, which promotes exudation in the surrounding tissue and brain edema development. Localized high intracranial pressure is caused by brain edema, which, in turn, causes localized ischemia and hypoxia, resulting in a vicious cycle of localized hypoxia and, ultimately, development of radiation brain necrosis [13–15].

**Fig. 1:**
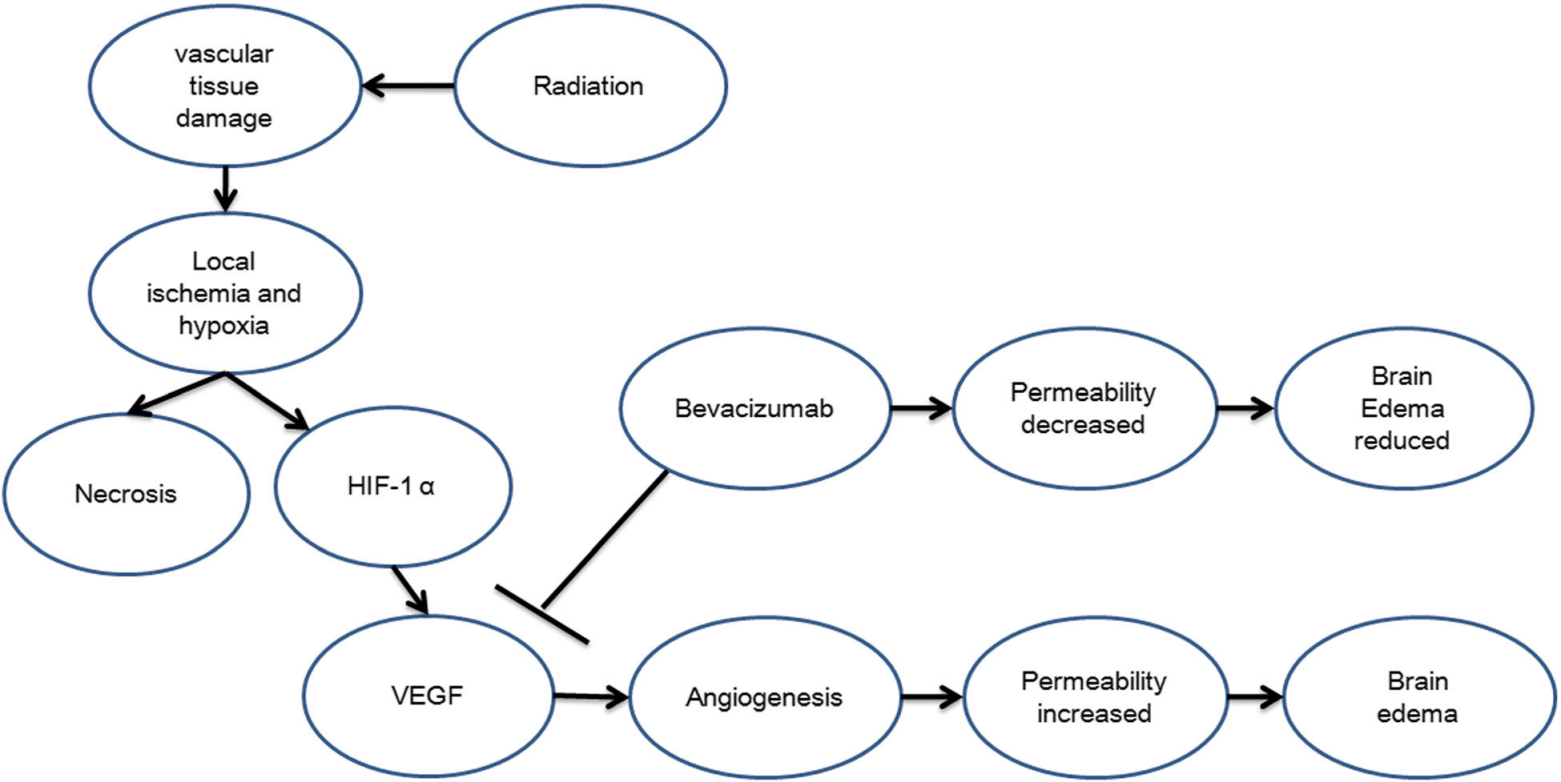
Mechanisms for bevacizumab treatment of radiation brain necrosis

A recombinant human monoclonal antibody, bevacizumab binds VEGF and prevents VEGF from binding its receptors (Flt-1 and KDR) on the endothelial cell surface, which plays a role in pruning blood vessels, regulating vascular permeability, reducing brain edema caused by brain necrosis and treating brain necrosis (Fig. 1). In addition, treating brain necrosis with bevacizumab features certain advantages over other anti-angiogenic drugs. First, for effective anti-angiogenic therapy, blood vessels must be treated with anti-angiogenic drugs for a long period of time. The long half-life (approximately three weeks) of bevacizumab is ideal. Second, bevacizumab is convenient to administer, allows for a relatively long dosing interval and does not require continuous use [15, 16].Therefore, bevacizumab is a targeted and advantageous drug for radiation brain necrosis.

However, the pathological change in necrotic tissue is irreversible, and fully necrotic brain tissue does not have blood vessels, which eliminates anti-angiogenic therapy. During brain necrosis treatment, bevacizumab targets the vessels around the necrotic area and can only alter a brain edema formed by new vessels, not necrosis. Therefore, the localized ischemia and hypoxia remain unchanged as long as the pathological basis for the necrosis remains. After bevacizumab is discontinued, HIF-1α expression might increase again in the tissue surrounding the necrosis, which re-forms the vicious cycleand eventually leads to brain necrosis recurrence.

### Efficacy of the bevacizumab treatment for brain necrosis

#### 2.1 Summary of studies on bevacizumab treatment of brain necrosis

In 2007, Gonzalez J [1] first reported on the efficacy of bevacizumab treatment for radiation brain necrosis, which remains an important trail-blazing study despite its small sample size. Since then, more than a dozen studies on using bevacizumab to treat brain necrosis have been published. However, clinical studies on brain necrosis differ from studies on cancer treatment because brain necrosis is an adverse reaction, and its incidence should be minimized in clinical treatments. As a result, radiation brain necrosis studies typically involve a small number of cases. In addition to several case reports, only approximately 9 studies have included more than 5 cases(Table 1) [1–9]. Based on these studies, although a pathological biopsy is the gold standard for diagnosing radiation brain necrosis, most cases are diagnosed based on imaging because obtaining a clinical biopsy is difficult. The bevacizumab dose is typically 5–10 mg/kg, q2-4w, and patients receive at least 2 doses. Bevacizumab shows good efficacy for improving a patient’s KPS score, symptoms and MRI imaging; further, its side effects are mild, and grade 3 (or above) side effects are rare. Many clinical studies have further established the clinical efficacy of using bevacizumab to treat radiation brain necrosis, which confirms a role for bevacizumab in treating radiation brain necrosis. Most studies show that bevacizumab exhibits good short-term efficacy for radiation brain necrosis; however, these studies feature the following drawbacks. ①Bevacizumab treatment was initiated immediately following a radiation brain necrosis diagnosis without investigating whether bevacizumab treatment of the necrosis was necessary. ②Screening was insufficient in certain cases, and good observations were impossible due to short survival in certain patients. ③The studies feature a short follow-up period and, in most cases, only short-term changes in radiation brain necrosis; neither development nor changes in long-term brain necrosis progression were observed. ④Relatively few studies have reported on bevacizumab resistance. Thus, the short-term efficacy of bevacizumab treatment for radiation brain necrosis has been established, but the treatment is not perfect, especially given a lack of long-term observations for radiation brain necrosis after bevacizumab is discontinued.

**Table 1:** The studies of bevacizumab treatment for radioactive cerebral necrosis

Year	Author	Cases	Diagnosis	Treatment schedule	Radiographic response (MRI)	Toxicity	follow-up time after last Bevacizumab delivery	Recurrence
2007	Gonzalez J et al.	8	MRI and biopsy	5 mg/kg/2 weeks or 7.5 mg/kg/3 weeks schedule	reduction in all 8 patients	NA	NA	NA
2009	Torcuator R, et al.	6	biopsy	10 mg/kg q2w × 6.8 average cycles	Radiographic response 6/6 patients (100%)	little	NA	NA
2009	Levin VA,et al.	14	MRI or/and biospy	7.5 mg/kg q3weeks × media 4 cycles	All Patients showed improvement in neurological symptoms or signs.	3/11 patients had serious adverse events	10 months	2 patients
2013	Furuse M, et al.	11	Amino Acid PET and MRI	5 mg/kg q2weeks × 3–6 cycles	The median reduction ratio was 65.5% of flair MRI.	little	NA	The 6-month and 1-year tumor or necrosis PFS rates were 81.8 and 36.4%
2013	Boothe D, et al.	14 lesions in 11 patients	MRI and PET, 2 patients had biopsy confirmed	10 mg/kg every 2 weeks. The mean duration was 96.2 days	RN volume decreased by a mean of 64.4%	NA	NA	7/14 patients recurrence.
2012	Wang Y, et al.	17	MRI and PET	7.5 mg/kg q2weeks × media 4 cycles	MRI imaging reduction of 54.9 and 48.4% in post-gadolinium and T2-weighted scans, respectively	little	median 6 months (4 to12 months)	1 patient
2014	Yonezawa S,et al.	9	MRI or MET-PET	5 mg/kg /2 weeks × 6 cycles	response in all patients	little	NA	NA
2015	Sadraei NH,et al.	24	MRI,PET and/or biopsy	5 or 10 mg/kg /2w, 7.5 or 15 mg/kg / 3w ×6 (2–13 cycles)	radiographic improvement in 23 of 24 patients	little	NA	NA
2016	Zhuang HQ,et al.	14	MRI,PET and/or pathology	5 mg/kg q3weeks × at least 3 cycles	12/14 patients MRI imaging reduction	little	median 10.0 months (1.2–38.0 months)	10/14 patients recurrence.
2018	Li Y,et al.	50	MRI	5 mg/kg every 2 weeks for up to 4 courses	24.0% patients did not have an effective response, and 76.0%patients showed an effective response	NA	6 months	NA
2018	Xu Y,et al.	112	MRI	5 mg/kg intravenously every 2 weeks, for 4 cycles	65.5% patients in the bevacizumab group showed response	NA	6 months	13 patients showed RN recurrence

*The tables only collected the clinical studies more than five cases. NA: no description in the paper

#### After bevacizumab discontinued, brain necrosis could be recurrence, and the pathological change in necrotic tissue is irreversible

As mentioned above, bevacizumab targets blood vessels around the necrosis area, not the necrosis; therefore, in theory, necrosis recurrence is inevitable. Many studies have reported brain necrosis recurrence after bevacizumab is discontinued [3–6, 17]. However, brain necrosis recurrence after treatment has clearly not attracted sufficient attention because nearly all studies have focused on bevacizumab efficacy, and only 1 case report focused on radiation brain necrosis recurrence [17]. Jeyaretna DS [18] provided an alternative explanation for 1 patient with radiation brain necrosis recurrence. One patient was treated for radiation brain necrosis with bevacizumab at 5 mg/kg, q2w, for 4 cycles. The patient initially showed significant improvement; however, an MRI scan performed 5 months after bevacizumab treatment began showed recurrence. The recurrence was considered related to excessive vessel pruning caused by excessive bevacizumab treatment, thereby aggravating the ischemia and hypoxia in the original brain necrosis area and exacerbating the brain necrosis. In our research, 14 patients have enrolled in this study to receive bevacizumab at 5 mg/kg, q3-4w, for at least 3 cycles (3–10 cycles). Among the 13 patients who responded to bevacizumab treatment, 10 patients presented radiation brain necrosis recurrence after bevacizumab was discontinued [19] (Table 1). Researchers have different opinions on the mechanisms underlying recurrence of radiation brain necrosis, and we believe that pathological changes due to necrosis are irreversible. Thus, once necrosis has developed, no medical treatment can regenerate brain tissue or make necrosis disappear. Further, as long as the pathological basis for necrosis remains, new vessels will reactively form around the necrosis area, and little can be done to change this pathological process.

In summary, the anti-angiogenic effects of bevacizumab are the basis for its mechanism of action. Bevacizumab reduces new vessel permeability and brain edema, which relieves brain necrosis symptoms, producing a good clinical outcome, addressing the patient’s problems and improving quality of life [20, 21].However, given the irreversibility of radiation brain necrosis or over-pruning of vessels around the necrosis area by bevacizumab and, thus, aggravation of localized ischemia and hypoxia, further exploration and attention are necessary to address radiation brain necrosis recurrence after bevacizumab is discontinued.

### Current issues in bevacizumab treatment of brain necrosis

#### Diagnosis of radiation brain necrosis: Is it a brain radiation necrosis?

Pathological diagnosis remains the gold standard for diagnosing radiation brain necrosis; however, many practical issues remain in clinical practice. First, for stereotactic radiotherapy, many brain tumors are close to the base of the skull or are located in important functional areas, which eliminate surgical resection as well as stereotactic biopsy and, thus, a pathological diagnosis. Second, few patients are willing to undergo biopsy after stereotactic radiotherapy. Third, a stereotactic biopsy may not provide a complete pathological picture of the tumor tissue. Moreover, it is difficult to ask patients with multiple intracranial metastases and who receive palliative treatment to undergo a craniotomy to confirm a diagnosis if brain necrosis is suspected, and, in such patients, a craniotomy is inconsistent with the treatment goal of prolonging survival and improving quality of life. Hence, although a pathological diagnosis is the gold standard for diagnosing radiation brain necrosis, it is difficult to implement in clinical practice. Thus, a comprehensive imaging modality is the most practical and common diagnostic method for radiation brain necrosis in clinical practice. Most studies have also used imaging diagnoses based on actual conditions in clinical practice [13, 21, 22]. However, notably, brain necrosis imaging changes must be monitored regularly, and various imaging methods should be used to confirm brain necrosis and differentiate brain necrosis from tumor recurrence. Further, a pathological diagnosis is still recommended (as applicable) in individual cases that are difficult to diagnose. Second, radiation brain necrosis needs to be differentiated from pseudo-progression after treatment. Pseudoprogression refers to an increase in the extent of new development or enhancement around the recently treated brain tumor. This image was initially similar to tumor progression, but improved or stabilized in follow-up images, mostly after temozolomide (TMZ) and radiation therapy. This reason is considered to be local inflammation caused by radiotherapy and chemotherapy, cerebral edema and transient permeability of the blood-brain barrier, leading to regional hyperenhancement. In imaging, thick and fluffy enhancements usually occur along the edge of the lesion, and the apparent diffusion coefficient (ADC) signal is higher and the cerebral blood volume (rCBV) signal is lower. Pseudo-progression usually occurs within 2 months after treatment, which is earlier than the typical period of radiation-induced brain necrosis after radiotherapy alone. Radiation-induced brain necrosis usually occurs 10 months after radiotherapy and is a late complication of radiotherapy. At the same time, radioactive brain necrosis is generally characterized by map-like enhancement on enhanced nuclear magnetics, accompanied by metabolic changes in spectral analysis, which are distinguishing features from pseudoprogression [23].

#### Indications for bevacizumab treatment of radiation brain necrosis: If it is a brain radiation necrosis, does it need treatment with bevacizumab?

All previous studies [1–13] have used bevacizumab immediately following a radiation brain necrosis diagnosis, and a question remains about whether this approach is appropriate. No studies have reported indications for bevacizumab treatment. Clarifying the treatment goal of radiation brain necrosis is the key to understanding bevacizumab treatment indications. Unlike tumor treatment, the goal for treating radiation brain necrosis is not prolonging survival but reducing symptoms and improving quality of life. Moreover, not all patients with radiation brain necrosis exhibit symptoms. Undoubtedly, symptomatic radiation brain necrosis requires treatment, but how should asymptomatic radiation brain necrosis (or after the symptom were controlled) be managed? Considering the bevacizumab treatment goal, the key indication for using bevacizumab is to treat radiation brain necrosis is symptoms. We recommend treatment in symptomatic patients only and monitoring asymptomatic patients, even if imaging suggests brain necrosis.

#### Optimization of bevacizumab administration: If it is treated with bevacizumab, how to use?

Optimizing bevacizumab administration is complex and involves dose, treatment course and criteria for discontinuation. First, regarding dose, in previous studies, researchers used different bevacizumab doses (2.5–10 mg/kg). Currently, the field has not produced a consensus on dose, and most studies have demonstrated that bevacizumab has good clinical efficacy [2–10, 12, 24].Certain researchers believe that higher doses are more effective at managing brain necrosis [7], but given the vascular mechanisms of brain necrosis and the features of anti-angiogenic therapy, we believe that treatment time is more important than plasma concentration. Moreover, we recommend low-dose bevacizumab in clinical practice due to the associated treatment cost. Regarding treatment course, in previous studies, patients typically received bevacizumab every 2-4 weeks for at least two doses (no maximum). Currently, the field has not produced a uniform standard. Because the bevacizumab treatment goal is symptom relief, not prolonging survival, we suggest that patients should be treated until symptoms are relieved and imaging improves; the treatment should then be discontinued and not used as a long-term treatment. For patients with recurrence, symptomatic patients should receive treatment, and asymptomatic patients as well as patients with long-term brain necrosis stability do not require treatment. Further, studies have reported anti-angiogenic drug resistance [25, 26]; however, currently, no studies have reported bevacizumab resistance in patients with radiation brain necrosis. For bevacizumab resistance, a question remains about whether bevacizumab should be discontinued (and the patient be monitored) and provided again upon progression or whether maintenance therapy should be provided following effective treatment of brain necrosis; clinicians should pay attention to this issue. Our case data show that re-treatment with bevacizumab was ineffective due to the potential for bevacizumab resistance upon brain necrosis progression following long-term bevacizumab use [27]. Moreover, JCO [18] reported that excess bevacizumab treatment may cause excessive vessel pruning, thereby aggravating localized ischemia and hypoxia of the necrosis area and resulting in brain necrosis recurrence. Hence, for cancer patients, bevacizumab treatment until brain necrosis progression may do more harm than good. Further, upon bevacizumab resistance, there is no available alternative to treat radiation brain necrosis, which yields inconsistent clinical treatments and affects clinical efficacy.

#### Prevention is the best treatment: How to avoid radiation brain necrosis?

Radiation brain necrosis is a complication; thus, the most important treatment is reducing the incidence of brain necrosis. A challenging issue for stereotactic radiotherapy is how well brain tissue tolerates large-dose radiotherapy. Currently, the field has not produced a consensus on the impact of tumor and treatment factors, such as treatment volume, tumor segmentation and tumor dose, on the incidence of brain necrosis [28, 29].Studies at the Tianjin Tumor Hospital show that the number of doses, whether whole-brain radiotherapy is used and radiotherapy BED are factors that affect the incidence of radiation brain necrosis. The receiver operating characteristic (ROC) curve shows that radiotherapy BED is the only good predictive factor for radiation brain necrosis. Based on the number of doses calculated from the threshold BED dose (> 7410 cGy) of radiation brain necrosis, and we also recommend the following in clinical practice [30]. In short, prevention is the best treatment, and using the appropriate prescribed dose based on history data and the patient’s condition is a key to reducing the incidence of radiation brain necrosis.

## Conclusions

In summary, bevacizumab prunes blood vessels, reduces nonvascular permeability in radiation brain necrosis and alleviates brain edema, thereby relieving the patient’s symptoms and improving quality of life [31–33]. However, necrosis is irreversible, and as long as the pathological basis for necrosis remains, new vessels will re-form and lead to brain necrosis progression. In addition, more clinical data are necessary to investigate indications for bevacizumab treatment of radiation brain necrosis, optimization for the mode of administration, bevacizumab resistance, and prevention and diagnosis of brain necrosis.

## Abbreviations

BED: Biological effective dose
HIF: Hypoxia inducible factor
MRI: Magnetic resonance imaging
VEGF: Vascular endothelial growth factor
